# Stiffness Gradients Mimicking *In Vivo* Tissue Variation Regulate Mesenchymal Stem Cell Fate

**DOI:** 10.1371/journal.pone.0015978

**Published:** 2011-01-05

**Authors:** Justin R. Tse, Adam J. Engler

**Affiliations:** Department of Bioengineering, University of California San Diego, La Jolla, California, United States of America; The University of Akron, United States of America

## Abstract

Mesenchymal stem cell (MSC) differentiation is regulated in part by tissue stiffness, yet MSCs can often encounter stiffness gradients within tissues caused by pathological, e.g., myocardial infarction ∼8.7±1.5 kPa/mm, or normal tissue variation, e.g., myocardium ∼0.6±0.9 kPa/mm; since migration predominantly occurs through physiological rather than pathological gradients, it is not clear whether MSC differentiate or migrate first. MSCs cultured up to 21 days on a hydrogel containing a physiological gradient of 1.0±0.1 kPa/mm undergo directed migration, or durotaxis, up stiffness gradients rather than remain stationary. Temporal assessment of morphology and differentiation markers indicates that MSCs migrate to stiffer matrix and then differentiate into a more contractile myogenic phenotype. In those cells migrating from soft to stiff regions however, phenotype is not completely determined by the stiff hydrogel as some cells retain expression of a neural marker. These data may indicate that stiffness variation, not just stiffness alone, can be an important regulator of MSC behavior.

## Introduction

In their niche, cells are presented with an array of complex biophysical and biochemical signals from the surrounding extracellular matrix (ECM) [1], [2], [3]. The Young's modulus, *E*, often referred to in a biological context simply as elasticity or stiffness, is an intrinsic ECM characteristic that has a profound effect on cell spreading, morphology, and function [4], [5], [6], [7]. In particular, stem cells show lineage-specific differentiation when cultured on substrates matching the stiffness corresponding to native tissue; neural stem cells become either neural or glial lineages depending on matrix elasticity [8], pre-osteoblasts most efficiently form calcified deposits when cultured on optimally stiff substrates [9], and multipotent mesenchymal stem cells (MSCs) [10] become neurogenic, myogenic, and osteogenic when cultured on substrates mimicking neural, muscle, and bone stiffness environments, respectively [11], [12], by regulating their cell tension [11], [13]. However these studies utilize polymer systems that have static parameters while their native counterparts reside in a dynamic environment in which elasticity may change spatially and/or temporally. For example, epicardial stiffness increases approximately 3-fold during development [14] while myocardium post-infarction forms a fibrotic scar that is 3- to 4-fold more stiff than surrounding muscle [15]. Elasticity also varies naturally at interfaces, e.g. hard, calcified bones are connected to soft cartilage [11], [16]. As MSCs egress from bone marrow and hone to these interfaces or migrate through tissue [17], they may encounter such stiffness gradient(s), and it is not clear whether the MSC response to these stimuli is to remain in place and differentiate, as with static materials [11], [12], or migrate in response to the stiffness gradient as with fibroblasts [18].

Several methods have developed *in vitro* elasticity gradients starting with polymerizing adjacent solutions of differing polymer concentrations to obtain a gradient at the solution interface [18]. More complex methods have employed microfluidic devices [19] or photolithographically-patterned photoactivated initiators [19], [20], [21], [22] to generate monomer and/or crosslinking density gradients. A hallmark of these studies is the observation that most somatic cells, e.g. fibroblasts, endothelial cells, and vascular smooth muscle cells [18], [19], [20], [21], [23], migrate in response to stiffness gradients in a process called “durotaxis,” with specific exceptions for cells originating from highly stratified structures [22]. However gradient strength, i.e. the degree of stiffness change per length, for these studies is typically in a pathological rather than physiological range [15]. A notable exception has shown that somatic cell migration is dependent on gradient strength, though the shallowest gradient – 10 kPa/mm – was still within a pathological range [24]. While some somatic cells may durotax in physiological gradients [20], each mature cell type exhibits lineage specific behavior within a physiologically relevant stiffness range [4], [6], [25].

On the other hand, undifferentiated MSCs lack such a preference and are in fact programmed by these surroundings [11], [12], [13]. Since much of their migration is likely to occur through tissue with physiological rather than pathological gradient(s) before reaching the site in need of regeneration, perhaps a more fundamental question is whether they durotax when presented with a physiological stiffness gradient <1 kPa/mm in the absence of other stimuli, e.g. soluble growth factor gradients which could induce chemotaxis. To better understand the role this potential signal could play in MSC fate, we cultured MSCs on a photopolymerized polyacrylamide (PA) hydrogel of varying stiffness and provide the first evidence that MSCs indeed appear to undergo durotaxis rather than remain stationary. Morphological and lineage marker assessment indicates that MSCs, even within shallow durotactic gradients, migrate to stiffer matrix and then differentiate into a more contractile cell, though this behavior is complicated by some degree of ‘memory’ of the previously soft environment from which they migrated.

## Results

### Surface Characterization of Gradient Hydrogels

**Figure 1:**
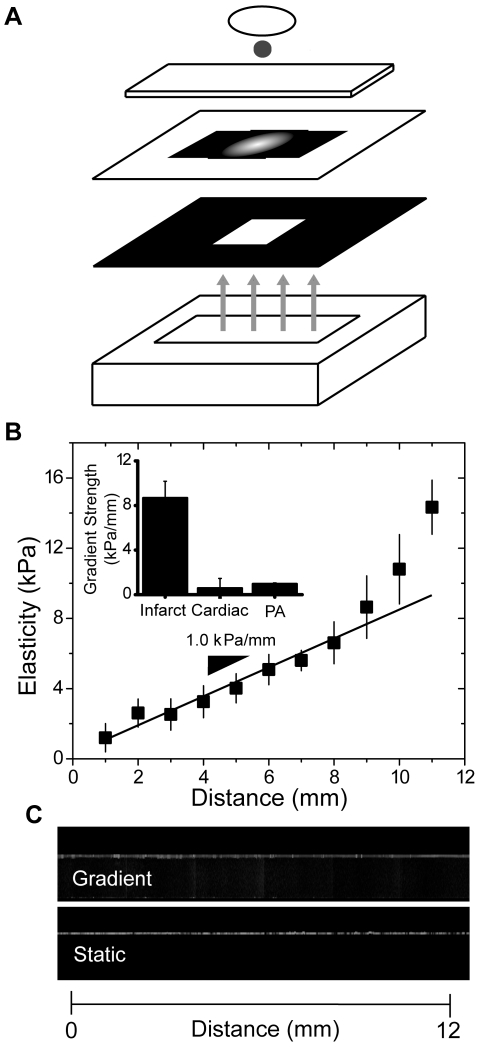
Hydrogel fabrication and characterization. (a) The schematic shows (from top to bottom) the 25 mm diameter aminosilanated glass coverslip, the acrylamide/bis-acrylamide solution with dissolved photoinitiator, a chlorosilanated glass slide, patterned photomask, negative photomask, and 254 nm UV light source. (b) AFM-determined elasticity of PA hydrogels was measured by every 1 mm from the center to the edge of the circular hydrogel. The gradient spans 10-fold change in elasticity from 1 to 14 kPa with a gradient strength of 0.96±0.12 kPa/mm. The inset shows a comparison of gradient strength of the hydrogel to those found in infarcted rat myocardium and the natural variations in adjacent, unaffected myocardium [15]. (c) Confocal microscopy images of collagen functionalized PA gradient (top) and static (bottom) hydrogels show collagen localized to the top surface of the hydrogels with roughly uniform distribution regardless of spatial changes in elasticity.

A photomask with a radial grayscale pattern was used to create a crosslinking gradient in a 10% acrylamide/0.3% bis-acrylamide hydrogel via selective activation of the photoinitiator Irgacure 2959 (Fig. 1A [26]). The elastic modulus with respect to distance from the edge to center of the hydrogels was measured by atomic force microscopy (AFM) and found to have a range of 1 to 14 kPa (Fig. 1B). Data was found to have a gradient strength of 1.0±0.1 kPa/mm. Such a gradient is within the physiological range of natural cardiac tissue variations, e.g. 0.6±0.9 kPa/mm, and considerably less than the pathophysiological range of infarct cardiac tissue, e.g. 8.7±1.5 kPa/mm, as previously measured [15]. To permit cell attachment, both gradient and static hydrogels were covalently functionalized with type I collagen using Sulfo-SANPAH, which showed relatively uniform attachment via antibody staining when observed in the XZ cross-section by confocal microscopy (Fig. 1C). Quantitative comparison of fluorescent intensity along the surface of the hydrogel indicated that any intensity variations were not statistically different (p = 0.87). Micron-sized antibody-bound beads were substituted for secondary antibodies to ensure that protein was surface accessible across the stiffness gradient and between static hydrogels of different stiffness or those with similar stiffness but different bulk polymer concentration (Fig. S1). Biochemical assessment of protein concentration (Fig. S1B inset) also demonstrated uniform bulk functionalization.

### MSCs Durotax to Stiffer Regions of the Gradient

**Figure 2:**
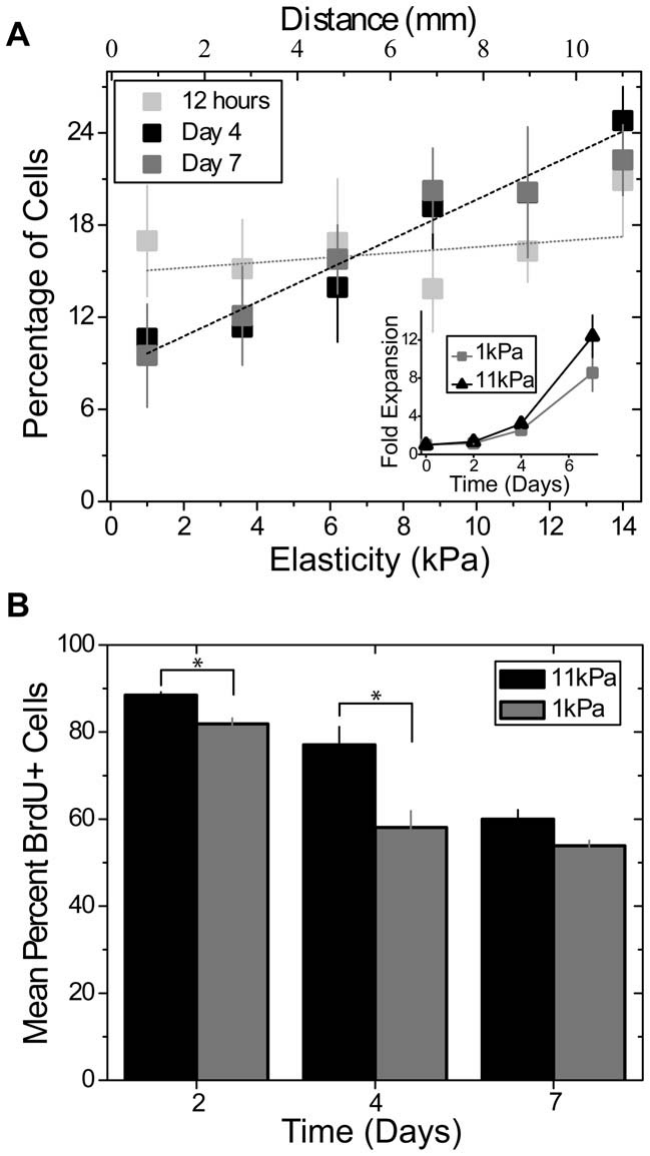
Migration and proliferation of MSCs on hydrogels. (a) At 12 hours, cell density is approximately uniform regardless of substrate stiffness (gray; p = 0.76 between data points). There was a loss and accumulation of MSCs on the softest and stiffest regions of the hydrogel, respectively, after both 4 and 7 days (p<0.005 and 0.05, respectively, using a one-way ANOVA comparison of slopes). The inset plot shows a noticeable proliferative difference in MSCs cultured on static hydrogels of 1 and 11 kPa. (b) BrdU staining of MSCs cultured on 1 or 11 kPa static hydrogels demonstrates that proliferation rate decreases as time increases for both stiffness values though MSCs on 11 kPa hydrogels proliferate at a slightly faster rate. *p<0.05 using student t-tests.

**Figure 3:**
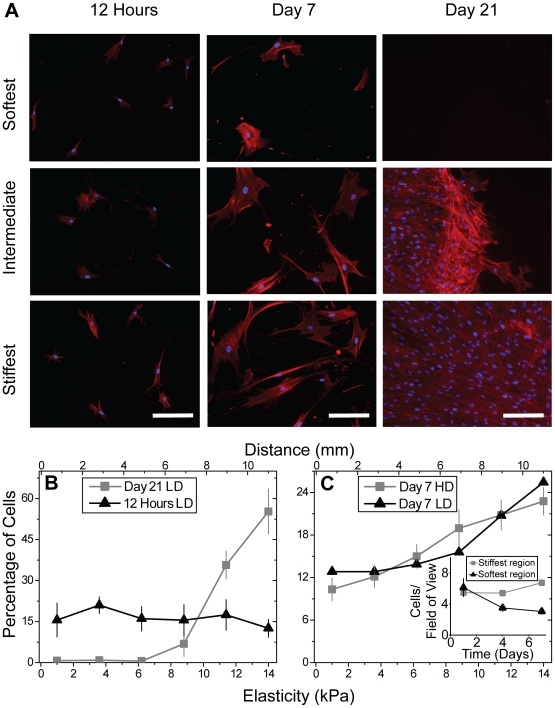
Spatial distribution of mitomycin C-treated MSCs on gradient hydrogels. (a) Images of Hoescht 33342 (blue) and phalloidin (red)-stained mitomycin C-treated MSCs plated at low density (250 cells/cm^2^) illustrate the change in distribution with time. After 21 days, MSCs are locally confluent in the stiffest region of the hydrogel. Scale bar is 56.5 µm. (b) At 12 hours, low density mitomycin C-treated cells were distributed evenly, while at day 21, essentially all the cells had migrated to the stiffest region of the hydrogel and formed a locally confluent layer. p<0.001 for all data comparing days 1 and 21. (c) At day 7, both low density and high (1000 cells/cm^2^) density cell seeding shows a 2-fold increase in cell density between the stiffest and softest regions of the hydrogel. The inset shows how the cell density changes at the stiffest versus the softest region over time. p<10^−2^ for both densities comparing cells at the center and edge.

Human mesenchymal stem cells (MSCs) were cultured on the collagen I-coated gradient hydrogels to determine whether MSCs will undergo directed migration on hydrogels or will differentiate in place. Cells were seeded at a low density (250 cells/cm^2^) to minimize cell-cell adhesion and traction forces transmitted to adjacent cells initially had a uniform spatial distribution, e.g. 12 hours. After 4 and 7 days of culture, cells' spatial distribution showed a 2-fold increase between the stiffest and softest regions of the hydrogel (Fig. 2A). Though nearly all cells remain viable as observed from calcein AM staining on soft and stiff static hydrogels (not shown), cell density (Fig. 2A inset) and proliferation rates–assessed by the percent of BrdU positive cells (Fig. 2B)–differed after 4 to 7 days in culture, which may explain why at the stiffest regions of the gradient, cells reached local confluency. To prevent proliferation and observe only durotaxis, MSC were pretreated with mitomycin C, a potent DNA crosslinker that prevents cell division, and allowed to migrate for up to 21 days. Again, MSC spatial distribution was biased towards the stiffest regions of the hydrogel after an initial uniform distribution when plated at low density (Fig. 3A). By 21 days, the center of the hydrogel became locally confluent (Fig. 3A, right), but given the mitomycin C treatment, this was created by all cells undergoing directed migration to the stiffest region of the hydrogel (Fig. 3B). Durotaxis can also be observed in mitomycin C-treated MSCs plated at higher densities, i.e. 1000 cells/cm^2^ [11] (Fig. 3C), and again a loss of cells at the softest regions and an accumulation of cells at the stiffest regions can be observed (Fig. 3C inset).

### Differentiation and Lineage Plasticity on Durotactic Gradients

**Figure 4:**
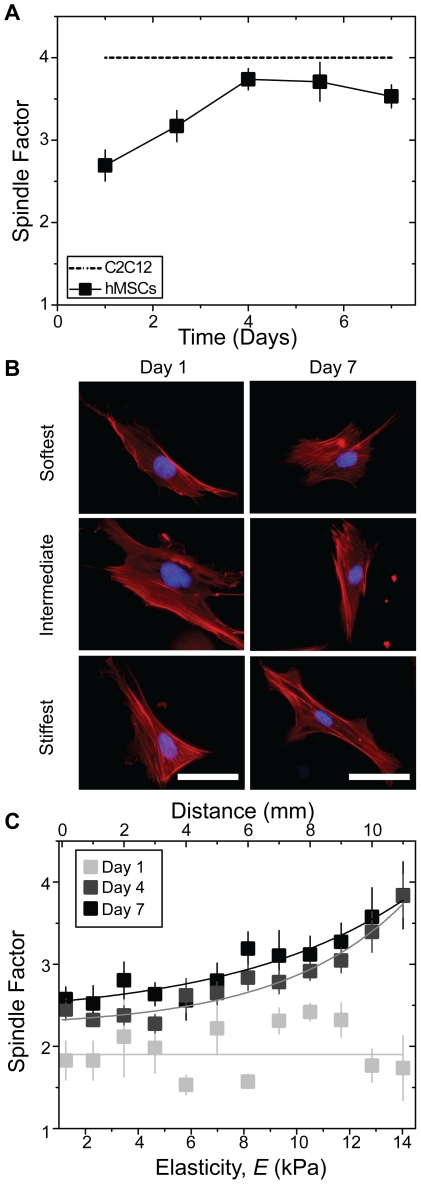
Morphological changes of MSCs as a function of time on gradient hydrogels. (a) MSCs cultured on static 11 kPa hydrogels increase their spindle factor whereas C2C12 myoblasts remain spindle-shaped throughout culture time as shown in [11]. (b) Morphological changes in cells stained with Hoescht 33342 (blue) and phalloidin (red) can be observed as a function of culture time and stiffness in MSCs cultured on gradient hydrogels. Scale bar is 12.5 µm. (c) Quantification of the spindle factor, i.e. the major divided minor axes, from images in (b) shows no relationship to stiffness at day 1, but by 4 and 7 days in culture, spindle factor increases from about 2.5 to 4 as a function of gradient stiffness.

**Figure 5:**
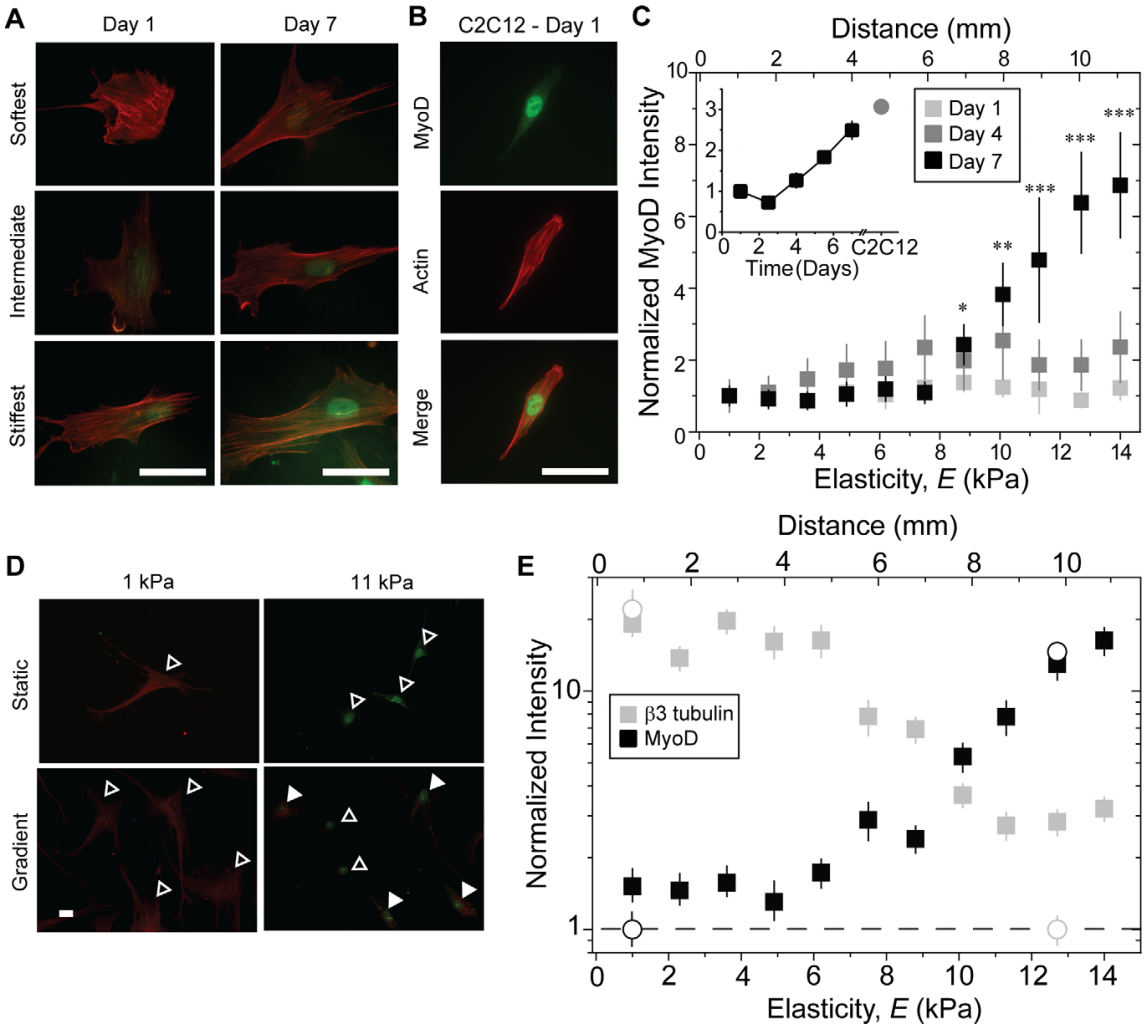
MSC expression of lineage markers as a function of time on gradient hydrogels. (a) Immunofluorescent staining for MyoD (green) and phalloidin (red) observed as a function of culture time and stiffness in MSCs cultured on gradient hydrogels. (b) Quantification of MSC nuclear immunofluorescent intensity over time and gradient position. Intensity was normalized to MSCs at the coverslip edge and shown as a fold change. Inset plot shows quantification of MSC nuclear immunofluorescent intensity (black squares) for cells cultured on static 11 kPa hydrogels over time. Nuclear immunofluorescent intensity of C2C12 myoblasts (gray circle) cultured for 1 day on static 11 kPa hydrogels is also shown. Intensity for all cells was normalized to MSCs at day 1. *p<10^−2^, ** p<10^−3^, *** p<10^−4^ compared to both days 1 and 4. (c) Immunofluorescent staining for MyoD (green) and phalloidin (red) in a C2C12 myoblast cultured for 1 day on a static 11 kPa hydrogel. (d) MSCs were cultured on 1 and 11 kPa static (top) and gradient (bottom) hydrogels and stained for β3 tubulin (red) and MyoD (green). Open arrowheads indicate cells expressing either β3 tubulin or MyoD while filled arrowheads indicate doubly stained cells. (e) MSC fluorescent intensity on gradient hydrogels (filled squares) was quantified for β3 tubulin (grey) and MyoD (black) and normalized to the non-permissive static hydrogel (open circles), i.e. β3 tubulin and MyoD intensities were normalized to MSC intensity on static 11 and 1 kPa hydrogels, respectively. The dashed line indicates no change from the non-permissive hydrogel of that protein. All scale bars are 12.5 µm.

MSCs on static 11 kPa hydrogels adopt spindle-shaped morphology by 4 days in culture (Fig. 4A), characteristic of C2C12 myoblasts [11], and subsequently express MyoD, a myogenic regulatory factor (see Fig. 5C, inset). MSCs on gradient gels are less polarized and randomly distributed initially but become spindle-shaped in a spatially-dependent manner after 4 days in culture (Fig. 4B, C). Spindle factor does not change between days 4 and 7 despite the accumulation of cells at the hydrogel's center, thus cell morphology may only reflect local absolute hydrogel stiffness as with smooth muscle cells [24]. Conversely, MSCs on gradients change durotactic speed the most over this time frame: the rate of change in MSC spatial distribution with respect to time peaks at 4 days in culture (Fig. S2). So to better examine cell fate, shifts in MSC lineage marker expression were monitored over time. For gradient hydrogels, some cells on the stiffest regions began to express MyoD between days 1 and 7 (Fig. 5A) with a nuclear localized staining pattern similar to C2C12 myoblasts (Fig. 5B). Unlike static hydrogels where MSCs begin to increase MyoD expression at day 4 (Fig. 5C inset), spatially-dependent MyoD expression increases in MSCs on gradient hydrogels only by day 7 (Fig. 5C). Together these data suggest greater durotaxis before expressing MyoD when a stiffness gradient is present.

While a myogenic phenotype is likely for cells that are always on stiffer regions of the hydrogel [11], [12], the vast majority of cells first durotax, and it is not certain if those cells display ‘memory’ of the soft region via continued expression of neural markers, e.g. β3 tubulin [11]. At day 7, 1 and 11 kPa static hydrogels show β3 tubulin and MyoD-positive MSCs, respectively, and cells remaining on soft regions of gradient hydrogels expressed β3 tubulin (Fig. 5D, open arrowheads). However, MSCs on the stiffer regions of the gradient displayed a mixed phenotype consisting of cells positive for MyoD alone (open arrowheads) and those also expressing low amounts of β3 tubulin (filled arrowheads). When β3 tubulin and MyoD fluorescent intensities were quantified and normalized to the non-permissive static hydrogel, i.e. 11 and 1 kPa hydrogels respectively, MSCs on stiffer regions had on average a 3-fold higher β3 tubulin fluorescent intensity versus the control static hydrogel. On the other hand, MSCs on softer regions had less than a 50% difference in MyoD fluorescence versus the control static hydrogel (Fig. 5E). Closer inspection of the distribution of β3 tubulin intensity indicates a degree of bimodality (Fig. S3), suggesting the existence of two cell populations.

## Discussion

The *in vivo* niche for MSCs is a complex array of biophysical and biochemical signals [1], [2], [3] containing numerous signaling gradients created by injury to which MSCs hone [17]. As MSCs traverse to through normal tissue, they must encounter physiological gradients, including stiffness [15]. Stiffness-induced differentiation is becoming well appreciated (see [4], [7], [27]), and when micropatterned, supra-physiological but spatially-controlled stiffness has been shown to regulate MSC position but not exclusively migration [28]. However it is not certain if or to what degree MSCs respond to shallow physiological stiffness gradients, i.e. 0.6±0.9 kPa/mm [15]. Here we presented MSCs with physiological stiffness gradients and demonstrated that an entire population will preferentially accumulate on stiffer hydrogel regions, regardless of cell seeding density. Concurrently during the fastest period of migration, i.e. day 4, cells displayed spatially-dependent morphology but did not show spatially-dependent changes in myogenic lineage marker expression as on static hydrogels. By day 7 however, MSCs showed spatially-dependent myogenic lineage marker expression despite residual expression of a neural fate in a subset of cells which may have first undergone directed migration.

The observation that a ‘differentiation hierarchy’ may exist, i.e. that there is greater durotaxis before MyoD expression, supports the idea that MSCs may be able to hone to injury sites using other mechanisms in addition to haptotactic [29] and chemotactic gradients [30], though an insoluble stiffness gradient is not likely to drive initial MSC egression from marrow. Once within the periphery of a tissue however, migration due to both chemo- and duro-tactic gradients are likely to be cooperative when the disease induces localized stiffening as with myocardial infarction [15]. On the other hand opposing gradients are unlikely *in vivo*, but MSC plasticity has been previously demonstrated using stiffness and growth factor cues of opposing lineages: MSCs on 1 kPa hydrogels challenged with either muscle or osteo-inductive media displayed a mixed phenotype after 1 week but were unaffected by the inductive media after 3 weeks [11]. Here we have tested plasticity using essentially two different stiffness values in the MSCs that migrated from soft to stiff regions of the gradient. We observed that markers characteristic of both myogenic and neurogenic lineages were expressed in a subset of the overall population leading to a bimodal distribution in β3 tubulin intensity. Should these doubly positive cells represent the durotactic fraction of the population, it would imply a degree of cell ‘memory.’ Though single cell migration and phenotype tracking using multiple fluorescently-labeled lineage marker proteins is perhaps ideal, especially considering the possibility of MSC ‘memory,’ these data here at least suggest that MSCs can remain plastic and express differentiation program(s) triggered by stiffness from a region in which they previous resided.

As with smooth muscle cells [24], these data show that MSC migration is independent of local hydrogel stiffness, i.e. regardless of where the cell is within the gradient, it continues to migrate towards the stiffer substrate. However, MSC fate is directly affected by local hydrogel stiffness and gradient range, e.g. 1–14 kPa. This range over which cells migrated is not likely to be physiological, i.e. the stiffness of healthy muscle only varies approximately between 8 and 15 kPa [4], [7], [27]. Moreover, multi-lineage MSCs *in vivo* do not occur as such large ranges within a tissue are unlikely; therefore the plasticity observed here may not be likely *in vivo*. On the other hand, *in vivo* gradient strength can at least range between 0.6 and 8.7 kPa/mm [15], and since we show here that MSC fate can be regulated even by a shallow gradient, it raises the question of whether MSC fate can be regulated by gradient strength. While many of the questions above can be investigated using this current gradient technique, i.e. increasing overall stiffness or adjusting the gradient by increasing UV cure time or changing the photomask gradient, respectively, it is important to note this method's limited stiffness range and gradient strength [26]. Microfluidic approaches to create gradients can provide a wider stiffness range, and gradient strength can be precisely tuned by microfluidic geometry [19], [21]. Regardless of the device, physiologically-appropriate gradient strength [15] and stiffness range is necessary [4], [7], [27]. Yet to more completely mimic pathological conditions, it may be appropriate to have a composite gradient that changes from physiological to pathological gradients, e.g. 0.6 to 8.5 kPa/mm, as does heart muscle post-myocardial infarction [15].

Two other critical aspects not accounted for in this gradient system are *in vivo* ECM structure and dimensionality. Matrix is naturally a fibrillar structure [1], [3] whereas the hydrogel is not. Natural ECM's alignment can significantly increase matrix stiffness anisotropically, i.e. create a 1D gradient, relative to one that is not organized or is not fibrillar [31]. Transglutaminases also stiffen matrix via crosslinking without significant increase in ligand density [32], but simple gradient increases in ligand density can also result in stiffness gradients [15]. While the specific mechanism *in vivo* is not certain, the 2D hydrogel here can sufficiently decouple these effects and illustrate the importance of durotactic considerations in therapies. This 2D system also may have significant predictive power for 3D behavior; within physiological ranges, 3D computational models and fibrillar collagen gels have illustrated durotactic increases coupled with haptotactic migration [33], [34], [35]. Stem cell stiffness-dependent differentiation also appears similar to 2D cases, though tension dependence is due to integrin ligation rather than spreading [36]. What these data perhaps indicate is that while the are subtle differences and coupling of different migration modes, ultimately 2D studies here provide substantial motivation to understand MSC homing to injury sites and their changes in phenotype along the way.

Perhaps one additional aspect that 2D models can easily provide is to improve our understanding of how cells sense stiffness and durotax, especially with such shallow gradients. To put MSCs' mechano-sensitivity in context, if the average spindle-shaped MSC is 40 µm long (see Fig. 4B) and perfectly aligned with the stiffness gradient, it will at most feel a difference of 40 Pa along its major axis. To durotax, an MSC must be sensitive enough to detect that small stiffness difference, which is at most only 4% of overall stiffness at the softest part of the hydrogels. Much of our current understanding of mechano-sensing comes from static hydrogels where stress fiber alignment has a non-monotonic relationship with stiffness [37] that parallels differentiation [11], [12] and in a tension-dependent mechanism [11], [13]: inhibition of myosin ablates the cell's response. For durotaxis specifically, directed migration has been successfully modeled by applying elastic stability theory to stress fibers under tension [38]. Although these data demonstrate that the actomyosin cytoskeleton maintains polarized morphology and requisite tension necessary for durotaxis, focal adhesion complexes at the leading edge of cells likely establish critical intracellular signaling gradients for durotaxis. For example, receptor-like protein tyrosine phosphatases [39] and focal adhesion kinase [40] have been implicated in mechanosensing at the leading edge of cells, and in a localized region of the cell, these proteins could undergo strain-induced conformational changes to expose binding sites beneficial to establishing intracellular signaling gradients [27]. Gradients of Rho activation [41] and calcium signaling may also be likely [18], but ultimately sensing may be a function of all of these mechanisms as well as others yet to be described. Moreover, it is important to note that while such tension-dependent mechano-sensing processes can occur in the absence of specific growth factors, MSC maintenance requires a non-trivial amount of serum [11], [12]. Other reports note that tension activates MSC responses to specific growth factors in serum-containing cultures [13]. Whether growth factors are required for durotactic sensing or whether they simply maintain cell survival during durotaxis is uncertain, but it is clear that tension is required for durotaxis [18].

Together though, migration and lineage specification data suggest that MSCs differentiate after undergoing durotaxis and that they also exhibit a degree of plasticity. While the *in vivo* presence of chemotactic and haptotactic gradients and the aphysical stiffness range investigated here may complicate the predictive ability of our data, these *in vitro* results at least complement previous infarction studies that show MSCs calcification 4 weeks post-injection into fibrotic muscle tissue [8] where large stiffness gradients are present [15]. These observations emphasize the importance of ECM properties as fundamental regulators of stem cell fate and demonstrate that known variation in these properties can have a profound affect on undifferentiated stem cell behavior.

## Materials and Methods

### Cell culture

Low passage number human MSCs (Lonza, Inc., Switzerland) were subconfluently cultured at 37°C, 5% CO_2_ in low glucose Dulbecco's Modified Eagle Medium supplemented with penicillin, streptomycin and 20% fetal bovine serum (Hyclone; Logan, UT). Cells were plated onto hydrogels at either 250 cells/cm^2^ except for comparisons with high cell density experiments where cells were plated at 1000 cells/cm^2^. Media change was performed every 4 days. To inhibit proliferation, the MSCs were treated with mitomycin C at 10 µg/mL for 3 hrs and rinsed three times with media before plating. The murine myoblast cell line C2C12 (ATCC) was cultured as a positive control in their normal growth media: 78% High Glucose DMEM+20% FBS+1% Chicken Embryo Extract+1% Penicillin/Streptomycin. C2C12 cell were maintained in their undifferentiated myoblast state and were not chemically induced to differentiate. All cell culture reagents and chemicals were obtained from Invitrogen (Carlsbad, CA) and Sigma-Aldrich (St. Louis, MO), respectively, unless otherwise noted.

### Preparation and Functionalization of Polyacrylamide (PA) Substrates

Polyacrylamide substrates with a uniform elasticity were prepared according to a previously established protocol by Pelham and Wang [5]. Briefly, solutions of varying acrylamide and bis-acrylamide concentrations were polymerized by ammonium persulfate (10% w/v; 1/100 v/v) and tetramethylethylenediamine (1/1000 v/v; Bio-Rad; Hercules, CA). The hydrogels were cast between a glass coverslip activated with 3-aminopropyltrimethoxysilane and a glass slide activated with dichlorodimethylsilane.

The polyacrylamide substrates with a gradient elasticity were prepared according to a previously established protocol by Tse and Engler [26]. Solutions of 10% w/v acrylamide, 0.3% bis-acrylamide were polymerized with a free radical photoinitiator, 0.5% Irgacure 2959 (1-[4-(2–Hydroxyethoxy)-phenyl]-2-hydroxy-2-methyl-1-propane-1-One; Ciba, Tri-Iso, CA), under a 254 nm UV light source through a photomask. The photomask was created using the gradient tool in Photoshop and printed at 1200 dpi on nitrocellulose film. Hydrogels were activated with a heterobifunctional crosslinker *N*-Sulfosuccinimidyl-6-(4′-azido-2′-nitrophenylamino) hexanoate (Sulfo-SANPAH) (Pierce; Rockfield, IL) in a two step reaction performed in non-amine containing HEPES buffer at pH 8.5. First, the nitrophenyl azide portion of the Sulfo-SANPAH was covalently bonded to amine groups within the polyacrlyamide surface upon activation with 365 nm UV light, outcompeting NHS groups for amines due to its promiscuity in binding. After significant washing with HEPES, 0.10 mg/mL type I collagen (BD Biosciences; San Jose, CA) in pH 8.5 HEPES buffer was incubated over overnight at 37°C to allow NHS groups to bind with the collagen. To assess the uniformity of the type I collagen coating, functionalized hydrogels were stained with monoclonal anti-type I collagen IgG (Sigma-Aldrich) and Alexa Fluor 546-conjugated secondary antibodies or 1 µm diameter Fluoresbrite carboxylate beads coated (Polysciences; Warrington, PA). A bicinchoninic acid (BCA) assay (Bio-rad) was also performed to measure total protein conjugated to the entire surface of each hydrogel.

### Characterization of Polyacrylamide Hydrogels

AFM was used to measure the elastic modulus at the nano-scale of both static and gradient hydrogels. Photopolyerized hydrogels were allowed to swell in water overnight before testing their mechanical properties by atomic force microscopy. Samples were placed on an Asylum 3D-BIO AFM (Asylum Research; Santa Barbara, CA) and probed with a pyramid-tipped cantilever (Olympus; Japan) having a nominal spring constant of ∼20 pN/nm as determined from thermal calibration. Samples were indented by the probe to yield force-indentation curves from which the elastic modulus, *E*, or stiffness was obtained using a Hertz cone model [42], [43], [44], fit up to 2 µm indentation. Samples were indented hundreds of times in a random pattern for static hydrogels and at known locations in a radial pattern for gradient hydrogels using an XY-piezoelectric motor-controlled stage to determine the rate of increase in modulus. To confirm a uniform coating of collagen I, stained samples were examined by a CARV II confocal microscope (BD Biosciences; San Jose, CA) mounted on a Nikon Eclipse TE2000-U microscope with a motorized, programmable stage using a CoolSnap HQ camera controlled by Metamorph 7.6 software. Image J software was used to quantify the relative fluorescent intensity of the attached type I collagen as a function of elasticity.

### Cell proliferation, viability, and assessment of Durotaxis

Overall cell distributions were determined for durotactic studies by assessing the spatial distribution of Hoescht 33342-stain nuclei using Image J software. 10 µM 5-bromodeoxyuridine (BrdU) was added to cell culture medium 12 hrs prior to fixation. Cells were washed once with PBS and then fixed in a solution of 3.7% formaldehyde in PBS for 15 minutes. Cells were then permeabilized with 0.1% Triton-X 100 (EMD Chemicals; San Diego, CA) for 15 minutes, treated with 1 M HCl for 30 minutes at room temperature, 2 M HCl for 30 minutes at 37°C, and blocked with 2% fetal bovine albumin in PBS for 60 minutes prior to staining with Alexa Fluor 647-conjugated mouse monoclonal anti-BrdU antibody overnight at room temperature followed by Hoechst 33342 (1∶10000) for 10 minutes at room temperature. To access cell viability, the cells were rinsed with PBS and stained with 0.25 µL calcein acetoxymethyl ester and 0.50 µL ethidium homodimer-1 in PBS for 30 minutes at 37°C.

### Lineage Specification Assays

For lineage specific proteins, cells were instead blocked with 2% ovalbumin in PBS and then stained using rhodamine phalloidin, Hoescht 33342, mouse monoclonal anti-MyoD IgG (Santa Cruz Biotechnology), and/or rabbit polyclonal anti-β3 tubulin (Sigma). Antibody detection was performed with Alexa Fluor 488, 546, and 647-conjugated secondary antibodies. All samples were examined by a CARV II confocal microscope (BD Biosciences; San Jose, CA) mounted on a Nikon Eclipse TE2000-U microscope with a motorized, programmable stage using a CoolSnap HQ camera controlled by Metamorph 7.6 software. Image J software was used to determine spindle factor [11], i.e. length of the cell's major divided by minor axes. Staining intensity of MyoD was also assess by Image J by thresholding the Hoescht 33342-stained nucleus image and using it as a mask on the transcriptional factor image to determine the integrated nuclear staining intensity. For β3 tubulin, a thresholded rhodamine-phallodin image was used to as a mask. Intensity was normalized to the negative control, i.e. static hydrogels for the lineage which was not induced at that stiffness, e.g. MyoD on 1 kPa. To aid in image presentation, image intensity for Fig. 5D was enhanced 2-fold in Image J, though original image intensity was used for quantification in Fig. 5E. With each measurement, n>100 cells from triplicate experiments.

### Statistical Analyses

All statistical analyses were performed using Origin 8.0 (Origin Lab, Northampton, MA). Differences among groups were assessed by ANOVA with Tukey's *post hoc* analysis to identify statistical differences among three or more treatments when p is at least less than 0.05. Differences between two treatments, as in Figs. 2B and S2, were assessed by Student's t-test to identify statistical differences when p is at least less than 0.05. All data is presented as mean ± standard error with each data point's x-value representing the average modulus or position for that image. Given image width, each point is ±0.4 mm or 0.4 kPa though error bars have been omitted for clarity of data presentation.

## Supporting Information

Figure S1 **Covalent collagen attachment is independent of gel composition.** (a) Composite images of micron-sized antibody-bound beads attached to type I collagen on stiffness gradient hydrogels (Gradient), static hydrogels of different stiffness (1 and 11 kPa), and those with similar stiffness but different bulk polymer concentration (1 kPa composed of the indicated monomer:crosslinker ratio). (b) Quantification of bead density per field of view. Inset shows bulk type I collagen density on the surface of each hydrogel as determined by BCA assay.
(TIF)

Figure S2 **Durotactic speed.** The rate of change of the MSCs' spatial distribution with respect to time, e.g. cell acceleration along the gradient, indicates that cells have the greatest change in durotactic migration at day 4. * p<10^−2^ for day 3 versus 4 and ** p<10^−3^ for day 4 versus 7 using student t-tests.
(TIF)

Figure S3 **Lineage marker ‘memory’ on gradient hydrogels.** The distribution of β3 tubulin immunofluorescent intensity in MSCs is plotted for specific regions of the gradient hydrogel. Average intensity, shown in Figure 5E, is displayed here as a line within each distribution.
(TIF)
