# Supplementary figures and images for: Stiffness Gradients Mimicking In Vivo Tissue Variation Regulate Mesenchymal Stem Cell Fate
Source: PLoS One. 2011 Jan 5;6(1):e15978. doi: 10.1371/journal.pone.0015978 (PMC3016411; doi:10.1371/journal.pone.0015978)

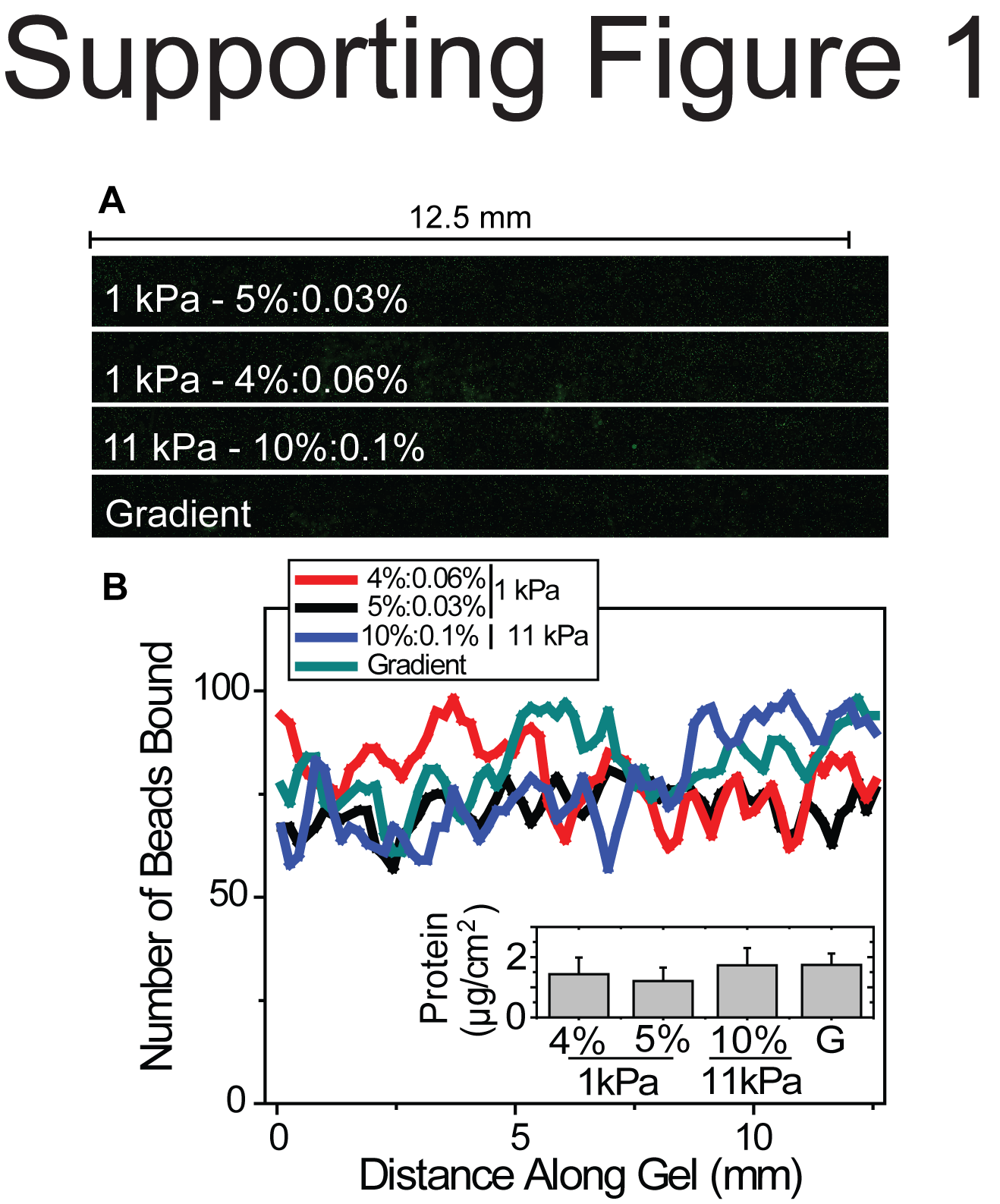

Supplement: Figure S1 — Covalent collagen attachment is independent of gel composition. (a) Composite images of micron-sized antibody-bound beads attached to type I collagen on stiffness gradient hydrogels (Gradient), static hydrogels of different stiffness (1 and 11 kPa), and those with similar stiffness but different bulk polymer concentration (1 kPa composed of the indicated monomer:crosslinker ratio). (b) Quantification of bead density per field of view. Inset shows bulk type I collagen density on the surface of each hydrogel as determined by BCA assay. (TIF) [file pone.0015978.s001.tif]

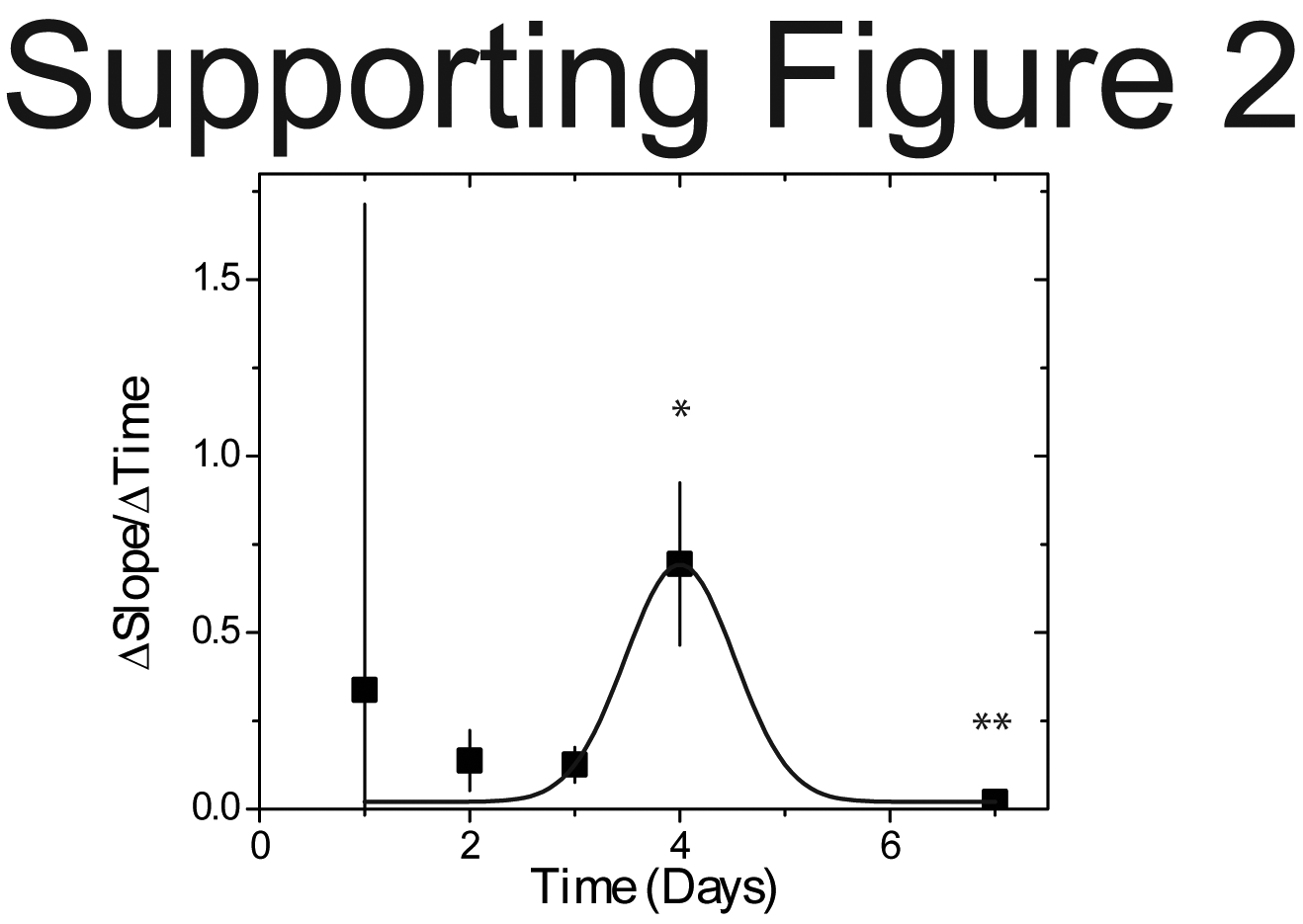

Supplement: Figure S2 — Durotactic speed. The rate of change of the MSCs' spatial distribution with respect to time, e.g. cell acceleration along the gradient, indicates that cells have the greatest change in durotactic migration at day 4. * p<10−2 for day 3 versus 4 and ** p<10−3 for day 4 versus 7 using student t-tests. (TIF) [file pone.0015978.s002.tif]

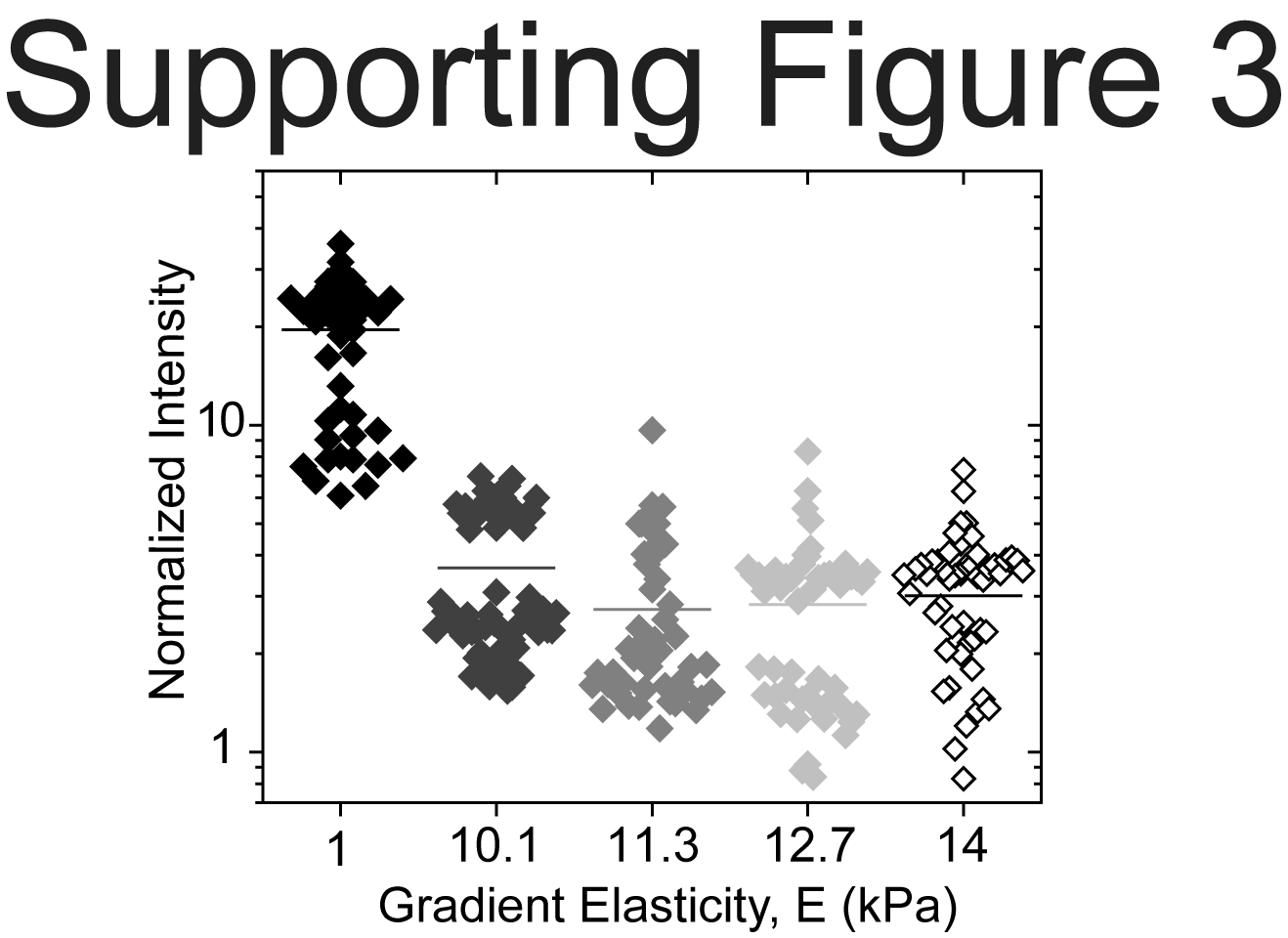

Supplement: Figure S3 — Lineage marker ‘memory’ on gradient hydrogels. The distribution of β3 tubulin immunofluorescent intensity in MSCs is plotted for specific regions of the gradient hydrogel. Average intensity, shown in Figure 5E, is displayed here as a line within each distribution. (TIF) [file pone.0015978.s003.tif]
